# Impact of Repetitive Transcranial Magnetic Stimulation (rTMS) on Brain Functional Marker of Auditory Hallucinations in Schizophrenia Patients

**DOI:** 10.3390/brainsci3020728

**Published:** 2013-04-29

**Authors:** Olivier Maïza, Pierre-Yve Hervé, Olivier Etard, Annick Razafimandimby, Aurélie Montagne-Larmurier, Sonia Dollfus

**Affiliations:** 1 CHU de Caen, Service Universitaire de Psychiatrie, Centre Esquirol, Caen 14000, France; E-Mails: olmaiza@yahoo.fr (O.M.); larmuriermontagne-a@chu-caen.fr (A.M.-L.); 2 Université de Caen Basse Normandie, UMR 6301 CNRS CEA, Centre Cyceron, Caen 14074, France; E-Mail: razafima@cyceron.fr; 3 Université de Bordeaux, GIN UMR 5296 CNRS CEA, Bordeaux 33076, France; E-Mail: pierre-yve.herve@u-bordeaux.fr; 4 CHU de Caen, Service d’Explorations Fonctionnelles du Système Nerveux, Caen 14000, France; E-Mail: etard-o@chu-caen.fr

**Keywords:** schizophrenia, auditory verbal hallucinations, functional magnetic resonance imaging, repetitive transcranial magnetic stimulation, voxel based morphometry, functional marker, language network

## Abstract

Several cross-sectional functional Magnetic Resonance Imaging (fMRI) studies reported a negative correlation between auditory verbal hallucination (AVH) severity and amplitude of the activations during language tasks. The present study assessed the time course of this correlation and its possible structural underpinnings by combining structural, functional MRI and repetitive Transcranial Magnetic Stimulation (rTMS). *Methods*: Nine schizophrenia patients with AVH (evaluated with the Auditory Hallucination Rating scale; AHRS) and nine healthy participants underwent two sessions of an fMRI speech listening paradigm. Meanwhile, patients received high frequency (20 Hz) rTMS. *Results*: Before rTMS, activations were negatively correlated with AHRS in a left posterior superior temporal sulcus (pSTS) cluster, considered henceforward as a functional region of interest (fROI). After rTMS, activations in this fROI no longer correlated with AHRS. This decoupling was explained by a significant decrease of AHRS scores after rTMS that contrasted with a relative stability of cerebral activations. A voxel-based-morphometry analysis evidenced a cluster of the left pSTS where grey matter volume negatively correlated with AHRS before rTMS and positively correlated with activations in the fROI at both sessions. *Conclusion*: rTMS decreases the severity of AVH leading to modify the functional correlate of AVH underlain by grey matter abnormalities.

## 1. Introduction

Auditory verbal hallucinations (AVH) are a frequent and disabling symptom in schizophrenia patients. They occur with a frequency estimated between 60% and 80%. In most cases, antipsychotic treatment can alleviate this symptom but in around 25%–30% of schizophrenia patients, AVH are resistant to pharmacological treatment [1]. 

The pathophysiology of AVH remains poorly understood even though the development of neuroimaging techniques has contributed to unveil some of the neurobiological underpinnings of this symptom. In the context of neuroimaging studies, two main strategies have been adopted so far. Some authors endeavored to capture the neural signature of the AVH by asking patients to signal the occurrence of AVH while they were being scanned (for meta-analysis see [2,3,4,5,6,7]). These studies evidenced that brain regions involved in speech processing are activated during AVH. The other approach, that can be complementary to the former, tries to identify functional markers of the propensity to hallucinate either by comparing cerebral activations during a cognitive task between patients with AVH and another population without AVH [3,4] or by uncovering correlations between the severity of the hallucinations and functional neuroimaging data [5,6]. These markers are independent of the actual occurrence of hallucinations during the scanning session but consist of brain areas whose function is modified in the context of a cognitive task in patients suffering from AVH. According to a recent meta-analysis, functional deficits in left temporal areas involved in auditory processing and speech perception may represent such markers [7]. The lower recruitment of left temporal areas during speech or auditory perception tasks in patients with AVH has been interpreted as indicative of a competition for neural resources between internally generated stimuli and outer speech [3], as if the cortex involved in speech processing was tonically tuned to process internal channels at the cost of processing external speech in patients with AVH [4]. 

These markers of the propensity to hallucinate deserve further characterization regarding their behavior over time. Indeed, since AVH severity often fluctuates along the course of schizophrenia, one might ask whether left temporal activations elicited by speech perception vary according to fluctuations of AVH severity. If the covariation between the severity of AVH and the cerebral activations in left temporal cortex were stable in time and were not affected by the fluctuations of AVH severity, it would strengthen the hypothesis of a causal link between functional activity in the left temporal cortex and hallucinatory behavior. In their seminal paper, Woodruff *et al.* [3] compared cerebral response to speech perception in schizophrenia patients while they were experiencing severe AVH and, 3 months later, after partial remission of AVH. They observed lower activations in temporal areas when AVH were severe, suggesting that cerebral activations follow the course of symptom severity. A limitation of this study, however, was the absence of a control group for taking into account a possible effect of the repetition of the same functional paradigm across two sessions. Indeed, in the context of longitudinal studies assessing functional biomarkers of schizophrenia, the question of the reproducibility of cerebral activations is crucial [8,9,10].

To investigate the time course of left temporal activations along with fluctuations of AVH severity, different strategies can be implemented. The easier but not the more efficient would be to scan patients at two different time points, expecting a spontaneous fluctuation of AVH severity. The main drawback of this approach is that it relies on the occurrence of a significant spontaneous change of AVH severity, what may fail to happen in a significant proportion of patients within a short period of time. Alternatively, a pharmacological intervention may induce a relief of AVH. Antipsychotics, however, are not only effective on AVH but target the whole spectrum of positive symptoms [11]. Another way would be to use repetitive transcranial magnetic stimulation (rTMS) which appears to be a promising tool to investigate biomarkers in schizophrenia. Moreover, rTMS over the left temporo-parietal area has been proposed recently as an add-on treatment for refractory AVH [12] and meta-analyses are in favor of a clinical efficacy of rTMS [13,14,15]. We recently reported promising results from a pilot study investigating the efficacy of high-frequency (20 Hz) rTMS on refractory AVH in schizophrenia patients [16].

Our aim in the present study was to characterize the time course of a putative functional trait marker of the propensity to hallucinate, namely the correlation between functional activation of the left temporal cortex and the severity of hallucinations. A subsample of Schizophrenia patients with refractory AVH who participated in our pilot study [16] received high frequency (20 Hz) rTMS and underwent two functional Magnetic Resonance Imaging (fMRI) sessions, before and after rTMS, of a story listening paradigm that has proven to yield reproducible activations across sessions [17]. A control group of healthy subjects underwent two fMRI sessions of the same functional paradigm without rTMS.

First, we identified the brain areas whose functional response correlated with the severity of AVH in schizophrenia patients before rTMS. Then, we studied the impact of the rTMS on the correlation between the severity of hallucinations and brain functional activations. Finally, since several previous studies reported structural correlates of AVH in left temporal areas [18,19,20,21,22,23], we investigated whether grey matter (GM) volume could underlie the functional marker of the propensity to hallucinate. 

## 2. Experimental Section

### 2.1. Participants

Eleven outpatients (six males, five females) with a diagnosis of schizophrenia (DSM-IV criteria established by the MINI plus) and 11 healthy participants matched one-to-one according to age, sex, handedness and level of education were recruited after giving informed written consent. The local Ethics board had approved the protocol. Data concerning the efficacy and tolerability of high frequency rTMS in this sample of schizophrenia patients were reported in a previous study [16]. Two patients, and their matched healthy controls, were excluded of the present study due to the absence of longitudinal fMRI data.

All patients had persistent AVH resistant to various pharmacological treatments and reported AVH the day before rTMS treatment. Six patients were taking second-generation antipsychotics, one was taking first-generation antipsychotics and two were taking both types of medicine.

Control participants were free of neurological and psychiatric disorders. Demographical and clinical characteristics of participants are given in Table 1.

**Table 1:** Sociodemographic and clinical characteristics of the study groups. AHRS, Auditory Hallucination Rating Scale; PANSS, Positive and Negative Symptom Scale; NA, not applicable.

	Schizophrenia patients (*n* = 9)	Healthy participants (*n* = 9)
Sex, M/F, *n*	4/5	4/5
Superior or equal 12 years of education, *n*	5/4	5/4
Age, mean ± SD (range), years	36.7 ± 10.7 (24–56)	37.8 ± 8.4 (26–50)
Handedness, right/left, *n*	8/1	8/1
Dose of antipsychotic (CPZ equivalent)	626 ± 598 (300–2150)	NA
Illness duration, mean ± SD (range), years	13.2 ± 9.7 (1.2–26)	NA
AHRS score at baseline, mean ± SD (range)	24.8 ± 4.5 (18–32)	NA
PANSS positive score at baseline, mean ± SD (range)	19.7 ± 4.8 (12–26)	NA
PANSS item P3 score at baseline, mean ± SD (range)	5.2 ± 0.6 (4–6)	NA
PANSS negative score at baseline, mean ± SD (range)	18.2 ± 4.9 (9–25)	NA
PANSS general psychopathology score at baseline, mean ± SD (range)	32.8 ± 7.6 (17–43)	NA
PANSS total score at baseline, mean ± SD (range)	70.7 ± 14.4 (56–91)	NA

### 2.2. FMRI Task Design

**Figure 1:**
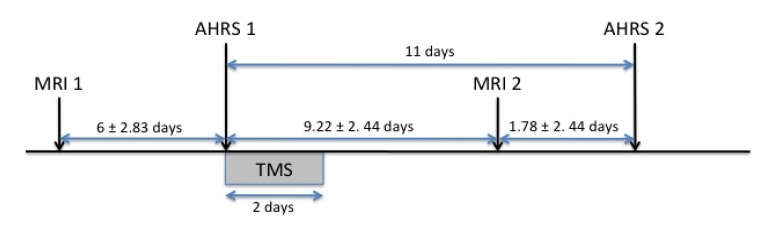
Time frame of the paradigm. MRI 1, first fMRI session; MRI 2, second fMRI session; AHRS 1, first AHRS assessment; AHRS 2, second AHRS assessment.

At each session, participants listened to the same factual story in French, interspersed with periods of rest. This paradigm showed good reproducibility in schizophrenia patients and in healthy subjects and the reproducibility of activations was not influenced by task performance [17]. Presentation of the stimuli followed a block design, with nine 30-s blocks: five blocks of rest were alternated with four blocks of the story. Participants were instructed to listen attentively to the story with their eyes closed. Both sessions were separated by an interval of 15.2 ± 3.38 days in patients and 14.8 ± 2.39 days in controls. The first session preceded rTMS stimulation by 6 ± 2.8 days (Figure 1).

### 2.3. Data Acquisition and Preprocessing

All MRI acquisitions were performed using a Phillips 3 Tesla scanner. During the anatomical session, structural T1-weighted images (sequence parameters: TR = 20 ms; TE = 4.6 ms; flip angle = 10°; inversion time = 800 ms; turbo field echo factor = 65; sense factor = 2; field of view = 256 × 256 × 180 mm; 1 × 1 × 1 mm^3^ isotropic voxel size) and T2-weighted multi-slice images (T2*-weighted fast field echo (T2*-FFE), sequence parameters: TR = 3500 ms; TE = 35 ms; flip angle = 90°; sense factor = 2; 70 axial slices; 2 × 2 × 2 mm^3^ isotropic voxel size) were acquired. At each functional session, T2-weighted structural images were acquired using the same sequence as during the anatomical session and functional images were acquired using a BOLD-fMRI T2*-weighted echo-planar sequence (repetition time = 2 s; echo time = 35 ms; flip angle = 80°; 31 axial slices; 3.75 mm^3^ isotropic voxel size). 

MRI data were preprocessed using Statistical Parametrical Mapping (SPM5; Wellcome Department of Cognitive Neurology, London, UK) and VBM 5.1 toolbox (Structural Brain Mapping Group, Christian Gaser, Department of Psychiatry, University of Jena, Jena, Germany) running on Matlab 7.4.

T1 images were iteratively segmented and normalized in Montreal Neurological Institute space using hidden random Markov fields to enhance signal to noise ratio. For subsequent Voxel Based Morphometry analysis, Grey Matter (GM) segments were modulated, using non-linear warps only. Of note, modulation with non-linear warping only corrects for non-linear deformation (local volume) so that correction for total intracranial volume is not needed in subsequent analysis. Then GM segments were smoothed with a 10 mm Gaussian filter.

The T2 images of the anatomical session were coregistered with T1 images, then T2 images of the functional sessions were coregistered with T2 images of the anatomical session.

Functional images were corrected for differences in time acquisition between slices and for head motion. They were coregistered with T2 images of the functional sessions and normalized using the parameters computed for normalization of T1 images. The normalized functional images had voxels of 2 × 2 × 2 mm^3^. They were subsequently smoothed with a Gaussian kernel of 8 mm and high-pass filtered (0.0078 Hz).

First level statistical analysis was performed applying the general linear model. The expected blood-oxygen level dependent (BOLD) signal change was modeled using a boxcar function convolved with a standard hemodynamic response function. The movement parameters from the realignment procedure were entered into the model as covariates. Individual contrast maps (French versus Rest) were computed for each subject and session.

### 2.4. Repetitive Transcranial Magnetic Stimulation

Only schizophrenia patients underwent rTMS. The Magstim high-speed magnetic stimulator, with biphasic current and a figure-of-eight coil (70 mm), air-cooled, was used. Thirteen trains of 200 pulses were delivered continuously over 10 s, with 50 s inter-train intervals at 20 Hz stimulation and 80% intensity of the resting motor threshold. Patients received rTMS twice a day (morning and afternoon, spaced by more than 3 h) for two consecutive days, for a total of 10,400 pulses. The stimulation site was fMRI guided according to a method described previously [16]. Briefly, patients underwent an fMRI language task and the rTMS stimulation site corresponded to the peak of highest activation along the posterior part of the left superior temporal sulcus.

### 2.5. Clinical Evaluations

Severity of AVH was evaluated with the Auditory Hallucination rating Scale (AHRS [24] before rTMS on the first day of treatment (AHRS1) and 11 days later (AHRS2) [16] (Figure 1). Patients’ symptomatology was also evaluated with the Positive and Negative Syndrome Scale (PANSS) [25] both before (PANSS1) and after (PANSS2) rTMS treatment.

### 2.6. Statistical Analyses

#### 2.6.1. Whole Brain Functional Analyses before rTMS

So as to identify brain regions whose activity was associated with the propensity to hallucinate, we searched for negative or positive correlation between the patients’ individual contrast maps and the AHRS scores at baseline through a SPM second level regression analysis (*p* < 0.001 uncorrected, *k* > 14 voxels corresponding to *p* < 0.05 at the cluster level). 

#### 2.6.2. Clinical Efficacy of rTMS

To assess clinical efficacy of rTMS, AHRS1 and AHRS2 scores were compared through a paired *t*-test. PANSS1 and PANSS2 total scores without the score at the item hallucination were also compared through a paired *t*-test.

#### 2.6.3. Time Course of the Link between Hallucinations and Cerebral Activations

To characterize the time course of the functional correlate of the propensity to hallucinate, we adopted a functional Region of Interest (fROI) approach. The significant cluster from the pre-rTMS analysis was defined as a fROI. We computed the correlation between mean individual signal variations in the fROI and AHRS scores after rTMS. 

Additionally, to evidence a potential effect of rTMS on cerebral activations in the fROI, mean individual signal variations were entered in a two-way mixed effect analysis of variance (ANOVA) with subject as a random effect and Group (patients *versus* controls) and Session (session 1 *versus* session 2) as fixed effects. Finally, we also computed the correlation across sessions of the mean signal variation in the fROI.

#### 2.6.4. Voxel Based Morphometry Analysis

In schizophrenia patients, the correlation between grey matter segment maps and AHRS scores at baseline was computed through a SPM regression analysis (*p* < 0.01 uncorrected, *k* > 700 voxels corresponding to *p* < 0.05 at the cluster level). Of note, we adopted the same threshold as Nenadic *et al*.’s study performing a similar analysis in schizophrenia patients [23].

To assess the link between structural and functional correlates of AVH, we computed the correlation between the grey matter volume in the significant cluster from the VBM analysis and the mean signal variations in the fROI at both sessions.

## 3. Results

### 3.1. Whole Brain Analysis before rTMS

**Figure 2:**
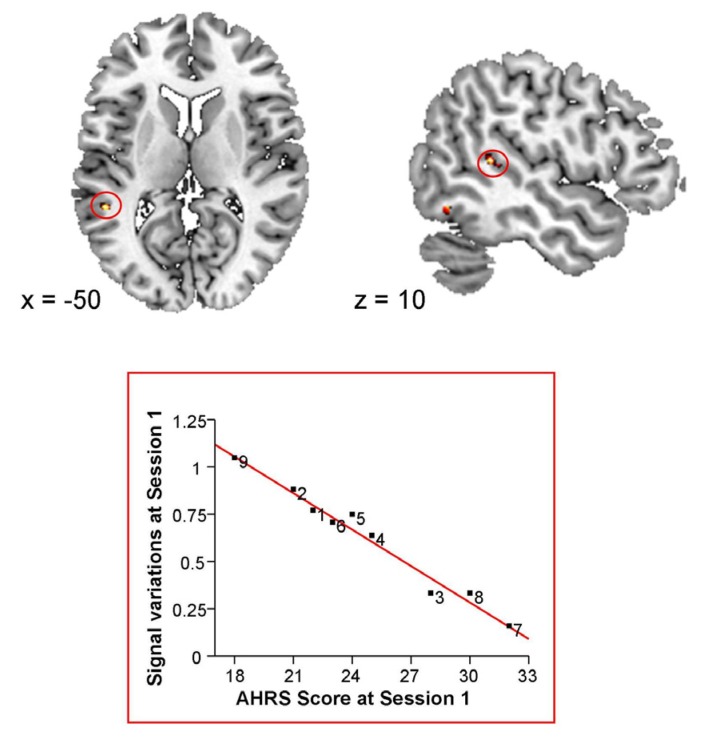
Correlation between baseline Blood Oxygen Level Dependent (BOLD) signal variations and baseline Auditory Hallucination Rating Scale (AHRS) scores.

The severity of hallucinations, assessed with the AHRS, was negatively correlated with brain activations in the posterior part of the left superior temporal sulcus (STS) (*x* = −50, *y* = −42, *z* = 10; 17 voxels; Figure 2).

Upper row: Results of the negative correlation between BOLD signal variations and AHRS scores at baseline in schizophrenia patients (*p* < 0.001 uncorrected, *k* > 14 voxels). Results are superimposed on a sagittal (*x* = −50) and axial (*z* = 10) slice of the single-subject Montreal Neurological Institute template. Bottom row: negative correlation between baseline mean signal variations in the significant cluster and AHRS scores at session 1. The numbers indexing patients are the same across all figures.

### 3.2. Clinical Outcome

The severity of auditory hallucinations significantly decreased after rTMS (AHRS1 score: 24.8 ± 4.5 (18–32); AHRS2 score: 12.2 ± 7.6 (0–20); two sample paired *t*-test: *t*(8) = 5.9, *p* = 0.0004; Cohen’s *d* = 2.16). The global symptomatology assessed with the PANSS also decreased after rTMS (PANNS1 score: 65.44 ± 13.10 (38–85); PANSS2 score: 57.67 ± 10.10 (42–70); two sample paired *t*-test: *t*(8) = 5.9, *p* = 0.03; Cohen’s *d* = 0.98).

### 3.3. Time Course of the Link between Hallucinations and Cerebral Activations

We observed no significant correlation between mean signal variations in the fROI at the second fMRI session and AHRS scores after rTMS (*r* = 0.28, *p* = 0.46). To ensure that the lack of significant correlation between cerebral activations and AHRS scores at session 2 was not related to our fROI approach, in the sense that the site of correlation between cerebral activations and AHRS scores might have shifted after rTMS, we conducted a whole brain regression analysis with functional scans of session 2 and AHRS scores at session 2. This analysis yielded no significant cluster (*p* < 0.001 uncorrected, *k* > 14 voxels).

**Figure 3:**
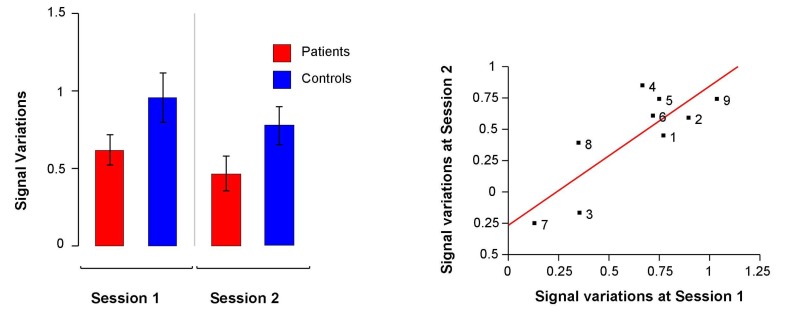
Left: Mean signal variations at baseline and at the second fMRI session in the functional Region Of Interest (fROI) in schizophrenia patients and in healthy participants. Right: Correlation between mean signal variations in the fROI at baseline and at the second fMRI session in schizophrenia patients.

The ANOVA performed on mean signal variations in the fROI detected a significant effect of session (*F*(1,16) = 14.94, *p* = 0.001), a trend for an effect of group (*F*(1,16) = 3.62, *p* = 0.07) but no significant group × session interaction (*F*(1,16) = 0.03, *p* = 0.85). In both groups, activations decreased at the second fMRI session and schizophrenia patients tended to activate this region to a lesser extent than controls (Figure 3).

Owing to the absence of a significant group × session interaction, we could not evidence a potential effect of rTMS. Finally, there was a strong correlation between mean signal variations in the fROI at both fMRI sessions (*r* = 0.82, *p* = 0.007; Figure 3).

### 3.4. Voxel Based Morphometry Analysis

**Figure 4:**
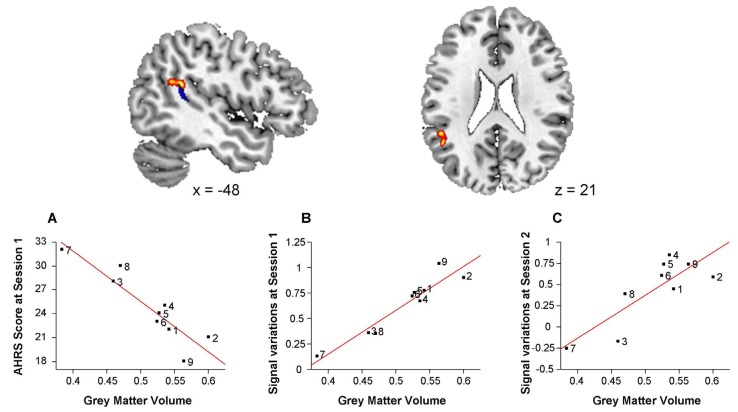
Negative correlation between grey matter volume and baseline Auditory Hallucination Rating Scale (AHRS) scores in schizophrenia patients.

In schizophrenia patients, grey matter volume was negatively correlated with AHRS1 score in a cluster located at the posterior part of the left superior temporal sulcus (*x* = −48, *y* = −44, *z* = 21, 757 voxels). This cluster was in the vicinity of the functional cluster from the baseline functional analysis. Mean grey matter volume in this cluster was positively correlated with mean signal variations in the fROI both at baseline (*r* = 0.95, *p* = 0.0001) and at the second session (*r* = 0.83 *p* = 0.006; Figure 4).

Upper row: In orange, cluster of significant negative correlation between grey matter volume and AHRS scores at baseline (*p* < 0.01 uncorrected, *k* > 700 voxels). In blue, significant cluster of the negative correlation between Blood Oxygen Level Dependent (BOLD) signal variation and AHRS score at baseline in patients, displayed at the same significance threshold as the cluster of the structural analysis. Note the partial overlap of the two clusters. Results are superimposed on a sagittal (*x* = −48) and axial (*z* = 21) slice of the single-subject Montreal Neurological Institute template. Bottom row: Correlation between mean grey matter volume in the structural significant cluster and AHRS scores at baseline (**A**), and mean signal variations in the fROI at baseline (**B**), and mean signal variations in the fROI after treatment (**C**).

## 4. Discussion

In this longitudinal study in schizophrenia patients with refractory AVH, we used an fMRI story listening paradigm and high frequency (20 Hz) rTMS to investigate the capacity of a functional marker of the propensity to hallucinate to reflect an experimentally-induced temporary decrease in the severity of AVH. Before rTMS we evidenced a significant negative correlation between AHRS scores and activations in a functional cluster, defined henceforward as a functional region of interest (fROI), located at the posterior part of the STS. After rTMS, this correlation was no longer significant. This decoupling between cerebral activations and hallucinations severity was explained by a significant decrease of AHRS scores following rTMS rather than by an effect of rTMS on cerebral activations in the fROI. Indeed, the ANOVA performed on cerebral activations in the fROI did not evidence significant group (patients *versus* controls) by session (session 1 *versus* session 2) interaction, and cerebral activations in the fROI were strongly correlated between sessions across subjects. Finally, grey matter volume in the posterior part of the STS was related both to baseline AHRS scores and to cerebral activations in the fROI. To our knowledge, this is the first study to evidence a relationship between functional and structural correlates of AVH. 

### 4.1. Baseline Results: A Functional Correlate of the Propensity to Hallucinate in the Posterior Part of the Left STS

Before rTMS, we observed a negative correlation between severity of hallucinations and cerebral activations in the posterior part of the left STS. Interestingly, this result is fully consistent with one study reporting, with a close neuro-imaging paradigm, a negative correlation between the severity of hallucinations and the activations in the posterior part of the left STS [5]. Moreover, when comparing patients with healthy subjects, we observed a trend for decreased activations in patients in this brain area. 

This finding is in line with the hypothesis of a competition for neural resources between internally-generated perceived speech and truly perceived speech as a possible mechanism at the origin of AVH. Indeed, in comparison with matched non-hallucinating patients, patients with AVH exhibit lower recruitment of auditory [4] and language related areas [3] when exposed to auditory or speech stimuli. Moreover, this lower recruitment is all the more pronounced as AVH are more severe [5,6].

### 4.2. Evolution of the Correlation between Cerebral Activations and Hallucinations

We investigated the impact of rTMS on a functional marker assessed by the correlation between the functional activity and the severity of hallucinations. After rTMS, this negative correlation was no longer significant suggesting a decoupling between AVH and their neurobiological correlates. Indeed, in line with our previous report [16], the scores at the AHRS dramatically decreased after rTMS pleading for a clinical interest of high frequency rTMS in the treatment of refractory AVH. In contrast, there was no evidence of an rTMS induced modulation of the magnitude of cerebral activations in the posterior part of the STS. Even if we observed a significantly lower activation in the fROI at the second fMRI session, it was common to both groups and might be ascribed to a learning effect [26]. This suggests that the rTMS intervention itself does not cause changes in BOLD response over and above the natural decrease in BOLD due to practice effects on the task.

Moreover, in spite of this aspecific session effect, activations within the fROI were strongly correlated across sessions. These results appear to contrast with a previous study [27] reporting increased cerebral activations following rTMS in the stimulated area. However, this study used a paradigm of word generation that is very sensitive to task performance and, crucially, did not include longitudinal data for the control group. Therefore, this variation of activation following rTMS could be linked to differences in performance across sessions.

This decoupling of symptoms and cerebral activations following rTMS has several implications regarding, on one hand, the neurobiological underpinnings of rTMS clinical efficacy and, on the other hand, the physiopathology of AVH. We were not able to evidence an impact of rTMS on cerebral activations in the left posterior STS beyond the session effect already observed in controls. Although we may have lacked sensitivity due to a limited number of patients, the brain signature of the clinical efficacy of rTMS may lie elsewhere. In this line, Vercammen *et al.* [28] reported an absence of rTMS modulation of the aberrant functional connectivity between the left temporo-parietal junction and the anterior cingulate cortex and amygdala that had been previously reported in patients with refractory AVH [29]. To explain this result, they proposed that rTMS may impact on the trigger of AVH (state characteristic) rather than on the propensity to hallucinate (trait characteristic). The contrast between the decrease of AHRS scores and the relative stability of cerebral activations in the fROI pleads against a straightforward causal link between a functional deficit in the posterior left STS and the severity of AVH since the covariation between cerebral activations and AHRS scores is not stable in time. Moreover, this finding goes against the above mentioned hypothesis of a competition for neural resources between hallucinations and truly perceived speech. [3,4]. Indeed, according to this hypothesis we would have predicted an increase of the functional activity in the STS in response to speech perception following the decrease of AVH severity. In other words, when a competitor (hallucinations) has left the arena, resources should be fully allocated to the one who remains, which is not the case. Our findings suggest that rTMS can induce a transitory change in the severity of AVH (a state change) whereas the functional activity in STS remains stable and might represent an enduring characteristic (trait) of the propensity to hallucinate.

### 4.3. Links between Functional Markers and Brain Structure

The baseline correlation between BOLD fMRI activations and hallucination severity contrasts with the stability of these activations across sessions despite a change in AHRS scores after TMS. A likely explanation would be a confounding effect of anatomical abnormalities. Indeed, the functional correlate of AVH that we evidenced is located in the posterior part of the left superior temporal gyrus, anatomical region that may be involved in the pathophysiology of AVH [18,19,20,21,22,23].

In this line, we evidenced a negative correlation between the grey matter volume in the posterior part of the left superior STS and the severity of hallucinations. The volume of the same region has been previously reported as being negatively correlated with the severity of hallucinations in a large sample of schizophrenia patients [23]. Crucially, the significant region of the VBM analysis partially overlaps the fROI where functional activity was negatively correlated to the hallucinatory score preceding treatment by rTMS. Accordingly, the functional activity in the fROI assessed at both fMRI sessions was positively correlated with the mean grey matter volume. Therefore, structural abnormalities in the patient group could constitute a third variable mediating the correlation between cerebral activations and severity of AVH. This would explain the fact that the BOLD signal variations at both sessions were correlated with the AHRS scores of the 1st session. These functional correlations would essentially reflect an underlying link between the anatomical abnormalities of the posterior STG region, potentially involved in the task, and the long-term propensity of the patient to hallucinate, irrespective of the severity of hallucinations after rTMS treatment.

When considered independently of the functional results, the results of our VBM analysis add to a growing literature on the importance of the macrostructural variability of the STG region in patients with AVH [18,19,20,21,22,23].The present observation further suggests that, in our sample of patients with refractory AVH, the patients’ AHRS scores before rTMS represent an enduring level of severity of AVH that can be transitorily modulated by therapeutic interventions (here, the rTMS) [30]. The results call for further investigations aimed at clarifying the relationships between the anatomy and the function of the posterior STG. This may require the use of other cognitive tasks and/or analytical tools able to separate functional variability from anatomical variability [31,32].

Finally, our results might reflect either a local reduction of cortical thickness or could be related to local variations of sulcal morphology [33]. Indeed, modifications of sulcal organization in temporal regions have been reported in schizophrenia patients with refractory hallucinations [34,35].

### 4.4. Limits

Our sample size was small as in previous studies combining neuroimaging and rTMS in schizophrenia patients with AVH [28,36]. Indeed, it is very challenging to include a large number of schizophrenia patients with refractory AVH and accepting to undergo rTMS as well as two sessions of MRI. However, both our functional and structural results replicated previous reports on the functional and structural correlates of the propensity to hallucinate [5,7,23]. Besides, we have chosen our functional paradigm because of its good reproducibility in both schizophrenia patients and healthy subjects [17]. Nevertheless, we observed a significant difference across sessions in both groups even if signal variations remained strongly correlated across sessions. This might be explained by a shorter intersession interval in the present study in comparison to our reproducibility study and by differences in MRI scanner across studies, the present one using a more sensitive 3 Tesla scanner. Finally, it is worth noting that our interpretation of a causal link between brain structure and functional activity as well as AHRS scores remains speculative. Indeed, we observed correlations between structure, functional activity and symptoms, which are not enough to infer causality.

## 5. Conclusion

In this longitudinal study, we used high frequency rTMS to investigate the link between the severity of AVH and a functional biomarker of the propensity to hallucinate. After rTMS, the baseline correlation between symptoms and cerebral activity was no longer significant. This was due to a decrease in the severity of AVH rather than to a rTMS-induced modification of brain activity. Our results suggest that functional abnormalities linked to the propensity to hallucinate could be underlain by structural differences and underscore the relevance of structural imaging in the interpretation of functional data. 

## Acronyms

AHRS: auditory hallucination rating scale
ANOVA: analysis of variance
AVH: auditory verbal hallucinations
BOLD: blood-oxygen level dependent
fMRI: functional magnetic resonance imaging
fROI: functional region of interest
Hz: hertz
PANSS: Positive and Negative Syndrome Scale
pSTS: posterior superior temporal sulcus
STS: superior temporal sulcus
rTMS: repetitive transcranial stimulation
